# Physiology-Related Variations in the Blood Hormone and Metabolome of Endangered Hog Deer (*Axis porcinus*)

**DOI:** 10.3390/metabo15020126

**Published:** 2025-02-13

**Authors:** Juan Wen, Bo Zhao, Yuqin Cao, Yu Qu, Liming Chang, Jie Mao, Yufei Li, Ruoyao Ni, Runliang Zhai, Jianping Jiang, Wei Zhu, Xuanzhen Liu

**Affiliations:** 1 Chengdu Zoo & Chengdu Research Institute of Wildlife, Chengdu 610081, China; juanwen881010@163.com (J.W.); cdzhaobo@126.com (B.Z.); yuqincao0627@163.com (Y.C.); quyu1120_hao@163.com (Y.Q.); cdzoomaojie@163.com (J.M.); cdzoo5156@163.com (Y.L.); 2 Chengdu Institute of Biology, Chinese Academy of Sciences, Chengdu 610213, China; changlm@cib.ac.cn (L.C.); niry@cib.ac.cn (R.N.); zhairunliang22@mails.ucas.ac.cn (R.Z.); jiangjp@cib.ac.cn (J.J.)

**Keywords:** biomarker, conservation, metabolomics, progesterone, testosterone

## Abstract

**Background/Objectives**: The hog deer (*Axis porcinus*) is an endangered species facing significant threats from habitat loss and fragmentation, with only captive populations remaining in China. Expanding breeding programs and restoring wild populations are critical strategies for the species’ conservation. Achieving this requires the development of an effective health database and the identification of molecular biomarkers for their physiological traits. **Methods**: In this study, we present the largest blood metabolomics dataset to date for captive hog deer, comprising 73 healthy individuals. We conducted targeted metabolomics to quantify blood hormone levels and untargeted metabolomics to characterize blood metabolic profiles, aiming to evaluate the associations of sex, age, and weight with metabolic profiles. **Results**: Our results reveal distinct growth patterns between females and males, with males reaching their body weight plateau at a larger size. We observed significant sex differences (*p* < 0.05) in blood hormones and metabolic profiles. Females exhibited higher levels of progesterone, hydroxyprogesterone, stress hormones (e.g., cortisol), and proline, while males had higher levels of testosterone, uric acid, phenylalanine, and guanidinosuccinic acid. Notably, body weight emerged as a more important factor than gender in explaining variations in the metabolome, particularly in males. Several blood biomarkers were identified as correlating with age and body weight. Specifically, blood progesterone levels in females were linked to both age and body weight, while in males, uric acid, prolylhydroxyproline, and 3-methylhistidine were associated with these factors. The potential significance of these results for the artificial breeding and conservation of hog deer were discussed. **Conclusions**: Our study provides a metabolic reference for identifying abnormal individuals and offers potential biomarkers for determining the gender, age, and body weight of hog deer. These findings may have significant implications for the artificial breeding and conservation efforts of the species.

## 1. Introduction

The hog deer (*Axis porcinus*), a member of the Cervidae family, comprises two subspecies: the nominate subspecies, found in India, Myanmar, Nepal, and Pakistan, and the Indochinese subspecies (*A. p. annamiticus*), native to Indochina, Thailand, and southern Yunnan, China [1,2]. Due to severe habitat loss, the global population of this species has experienced a dramatic decline, leading to its classification as Endangered on the IUCN Red List [3]. In China, the Indochinese subspecies was first identified in the late 1950s, primarily along the Nanding River in Gengma and Ximeng counties, near the China–Myanmar border in Yunnan. However, extensive agricultural development during the 1960s caused significant ecological degradation, resulting in the species becoming exceedingly rare in the wild. By the 1970s, the wild population of hog deer in China was presumed extinct, with only around 20–30 individuals remaining in captivity [4,5,6]. Today, the hog deer is one of the rarest deer species and is listed as a Class I protected animal under China’s List of State Key Protected Wild Animals. To ensure its survival, expanding breeding programs and re-establishing wild populations are critical conservation priorities.

Efforts in the artificial breeding of hog deer have made notable progress in recent years [7], increasing the captive population to approximately 145 individuals. This success is attributed to an improved understanding of the species’ reproductive biology and physiology [8]. For instance, accumulated dietary data on fawns have provided critical insights for precise nutritional assessments [8], while analyses of deceased hog deer have revealed correlations between vitamin and mineral levels and survival rates [9]. However, two key challenges must be addressed to ensure the successful reintroduction of captive hog deer into the wild. The first is a comprehensive investigation of the genetic diversity and structure of the species [10], which is crucial for guiding artificial breeding programs and enhancing the genetic variability of the population [11,12]. The second is the establishment of an effective health database for hog deer [13], alongside the identification of key molecular markers for rapid evaluation or prediction of physiological traits (e.g., sex and age), health status, and even mortality risks in both captive and wild populations.

The use of blood biomarkers in wildlife monitoring and health assessment offers significant potential [14]. For instance, biochemical markers have been successfully used to evaluate the health of sea turtles, serving as diagnostic tools to guide decisions on overall care and specific treatments during rehabilitation [15]. Research in this area for hog deer remains limited. Liu et al. conducted a comparative study on the blood transcriptome of hog deer, identifying several mRNAs associated with sexual dimorphism [16]. Compared to mRNA, hormones and metabolic molecules in the blood may hold greater promise as biomarkers for physiological health [17]. Hormones serve as upstream regulators of physiological and metabolic pathways, exerting a significant influence on the animal’s physiological state. Sex hormones, for example, provide insights into reproductive capacity and can inform population dynamics, while stress hormones reflect an individual’s stress levels, offering valuable information to optimize captive breeding conditions and understand the survival challenges faced by wild populations. Metabolites, as the end products of cellular processes, act as effectors of biological activity and directly reflect an individual’s physiological state. Therefore, we may anticipate associations between hormone levels, metabolic profiles, and the physiological state of hog deer. Currently, our understanding of the hormone and metabolite profiles in the blood of hog deer is limited. By integrating physiological data such as gender, age, and health status from captive individuals, we can establish a comprehensive database of blood hormones and metabolites. This approach would facilitate the identification of biomarkers associated with gender, age, and health, thereby supporting artificial breeding programs and assisting in the successful reintroduction of hog deer into their natural habitats.

In this study, we conducted the largest blood-based research on hog deer to date, relying on the Chengdu Zoo, which houses the largest captive population (96 individuals) of this species [18]. We employed quantitative targeted metabolomics to analyze the sex hormone and stress hormone profiles of 73 individual hog deer, alongside untargeted metabolomics to explore their blood metabolite profiles. By integrating data on gender, age, and body weight, we examined sex differences in hormone and metabolite profiles and identified several blood biomarkers associated with these factors. This research lays the groundwork for developing a comprehensive health database for hog deer.

## 2. Materials and Methods

### 2.1. Animals and Sample Collection

A total of 96 hog deer are maintained at Chengdu Zoo, Chengdu, China. These hog deer are fed twice daily, at 10 a.m. and 4 p.m. Their diet consists of concentrated feed, abundant fresh grass, hay, and a small number of fresh leaves, with no fixed proportions for these components. The deer are housed across three separate enclosures, each subdivided into multiple pens. Each pen contains one or more individuals, typically related by kinship or mating. Under the supervision of a professional veterinarian, the hog deer were anesthetized with Zoletil, an injectable anesthetic containing Tiletamine and Zolazepam, prior to blood collection.

Blood samples were collected using EDTA anticoagulant tubes and processed promptly to ensure efficient plasma separation. Plasma was separated by centrifugation at 3000 rpm for 10 min at room temperature. Samples left at room temperature were centrifuged within 1 h of collection, while those stored on ice were processed within 2 h. Following centrifugation, the plasma was carefully transferred into 1.5 mL centrifuge tubes in 0.2 mL aliquots and stored at −80 °C. A total of 73 individuals (female:male = 41:32) were collected for their blood samples, with ages ranging from 2 to 19 years (1–5, 38 individuals; 6–10, 25 individuals; 11–15, 7 individuals; 16–20, 3 individuals). Each animal was sampled for blood only once. More information was detailed in Supplementary Data S1: Table S1.

### 2.2. Targeted Metabolomics

Targeted metabolomics were employed to quantify hormone levels in the blood of hog deer. A total of 73 blood samples were analyzed, representing 73 distinct individuals. The analyzed hormones included corticosterone, cortisol, cortisone, 11-deoxycorticosterone, 11-deoxycortisol, aldosterone, estrone, estradiol, estriol, progesterone, testosterone, stanolone, androsterone, dehydroepiandrosterone, and melatonin. For each sample, 100 μL of blood was aliquoted and mixed with 150 μL of pre-chilled methanol and 1000 μL of pre-chilled methyl tert-butyl ether. The mixture was vortexed and incubated at 4 °C with shaking for 60 min, followed by 15 min of ultrasound-assisted extraction in an ice bath. Subsequently, 100 μL of pure water was added, and the mixture was vortexed and left to rest at room temperature for 10 min. The samples were then centrifuged at 16,000× *g* at 4 °C for 20 min. The supernatant was collected and dried using a high-speed vacuum concentrator.

For mass spectrometry analysis, the dried samples were reconstituted in 50 μL of a dichloromethane/methanol solution (1:1, *v*/*v*). The reconstituted solution was centrifuged at 20,000× *g* at 4 °C for 15 min, and the supernatant was collected for analysis. Sample separation was performed using a Nexera X2 LC-30AD ultra-high-performance liquid chromatography system (Shimadzu, Kyoto, Japan). The mobile phases consisted of 0.1% formic acid in water (A) and 100% acetonitrile (B). Samples were maintained at 4 °C in an autosampler, while the column temperature was set to 40 °C. The flow rate was 300 μL/min, with an injection volume of 5 μL. A gradient program was applied as follows: 0–5 min, B increased from 20% to 65% (linear gradient); 5–7 min, B increased from 65% to 95% (linear gradient); 7–10 min, B held at 95%; 10–10.1 min, B decreased from 95% to 20% (linear gradient); 10.1–13 min, B maintained at 20%.

Mass spectrometry was conducted using a 6500+ QTRAP mass spectrometer (AB SCIEX, Framingham, MA, USA) in positive ion mode. The ESI source parameters were as follows: source temperature, 550 °C; ion source gas 1, 40 psi; ion source gas 2, 50 psi; curtain gas, 35 psi; ion spray voltage, 5500 V. Multiple reaction monitoring (MRM) mode was used to detect the targeted ion pairs. Chromatographic peak areas and retention times were extracted using MultiQuant software 2.0.2. Retention times were calibrated using hormone standards, and metabolite identification was confirmed. Hormone concentrations were quantified using standard curves generated from hormone standards. System stability was assessed by comparing the base peak chromatograms of quality control (QC) samples, ensuring the reliability and consistency of the experimental workflow. The hormones concentrations were provided in Supplementary Data S1: Table S2.

### 2.3. Untargeted Metabolomics

Untargeted metabolomics was employed to characterize the metabolic profile in the blood of hog deer. A total of 73 blood samples were analyzed, representing 73 distinct individuals. For each sample, 100 μL of blood was transferred to an EP tube, followed by the addition of 400 μL of pre-chilled methanol. The mixture was vortexed and subjected to ultrasonic extraction in an ice bath for 20 min. Afterward, the samples were incubated at −20 °C for 1 h and centrifuged at 16,000× *g* for 20 min at 4 °C. The supernatant was collected and dried using a high-speed vacuum concentrator. The dried samples were reconstituted with 100 μL of pre-chilled methanol–water (1:1, *v/v*) solution. The reconstituted solutions were centrifuged at 20,000× *g* for 15 min at 4 °C, and an appropriate volume of the supernatant was collected for analysis. Samples were maintained at 4 °C in an automatic sampler during the analysis.

Chromatographic separation was carried out using a SHIMADZU-LC30 ultra-high-performance liquid chromatography (UHPLC) system equipped with an ACQUITY UPLC^®^ HSS T3 column (2.1 × 100 mm, 1.8 µm, Waters, Milford, MA, USA). The injection volume was 4 μL, the column temperature was set to 40 °C, and the flow rate was maintained at 0.3 mL/min. The mobile phases were 0.1% formic acid in water (A) and 0.1% formic acid in acetonitrile (B). The gradient elution program was as follows: 0–2 min, B = 0%; 2–6 min, B increased linearly from 0% to 48%; 6–10 min, B increased linearly from 48% to 100%; 10–12 min, B held at 100%; 12–12.1 min, B decreased linearly from 100% to 0%; 12.1–15 min, B held at 0%.

Mass spectrometry analysis was performed using a QE Plus mass spectrometer (Thermo Scientific, Waltham, MA, USA) with a heated electrospray ionization source, operating in both positive (+) and negative (−) ion modes. The ionization settings were as follows: spray voltage, 3.8 kV (+) and 3.2 kV (−); capillary temperature, 320 °C; sheath gas flow rate, 30; auxiliary gas flow rate, 5; probe heater temperature, 350 °C; S-lens RF level, 50. Data acquisition spanned 15 min with the following parameters: full-scan range, 75–1050 *m*/*z*; resolution for full MS, 70,000 at *m*/*z* 200; automatic gain control target, 3 × 10^6^; maximum injection time (IT) for full MS, 100 ms. For MS2 analysis, the 10 most intense precursor ions from each full scan were selected. The MS2 acquisition parameters were as follows: resolution, 17,500 at *m*/*z* 200; automatic gain control target, 1 × 10^5^; maximum IT, 50 ms; isolation window, 2 *m*/*z*; stepped normalized collision energy, 20–30–40; activation type, HCD.

Raw data were processed using MSDIAL software 4.80 for peak alignment, retention time correction, and peak area extraction. Metabolite identification was performed through accurate mass matching (mass tolerance < 10 ppm) and secondary ion spectrum matching (mass tolerance < 0.01 Da) against public databases, including HMDB, MassBank, GNPS, and an in-house metabolite library (BP-DB). The system’s stability was evaluated by comparing the base peak chromatograms of QC samples to ensure the reliability and consistency of the experimental workflow. The metabolite abundance matrix was provided in Supplementary Data S2: Table S3.

### 2.4. Statistical Analyses

Basic statistical analyses were conducted using IBM SPSS v21.0 (IBM, Armonk, NY, USA) and R [19]. The Kolmogorov–Smirnov and Shapiro–Wilk tests were employed to assess whether the data significantly deviated from a normal distribution, and Levene’s tests were conducted to evaluate homoscedasticity across groups. The sexual differences in body weight and age were analyzed using Mann–Whitney U test. The overall similarity in hormone profile and metabolome between samples were displayed using principal component analysis (PCA, based on metabolite abundance matrix) and principal coordinates analysis (PCoA, based on Bray–Curtis distance of the metabolite abundance matrix). Single- or multi-factor PERMANOVA were performed to examine the inter-group differences in hormone profile and metabolome (using type II sums of squares) [20]. Analysis of covariance (ANCOVA) were performed to identify the metabolites that differ between genders, with gender as fixed factor and body weight or age as covariates, and the *p* values were adjusted with Benjamini–Hochberg (BH) corrections. Spearman and Pearson correlations were performed to identify the potential age or weight biomarkers, followed by BH correction to adjust *p* values. Cytoscape was used to construct correlation networks. Graphs were generated using GraphPad Prism 5, MedPeer (www.medpeer.cn, accessed on 20 October 2024), and ggplot2 [19].

## 3. Results

### 3.1. Sex Differences in Physiological Characteristics

**Figure 1:**
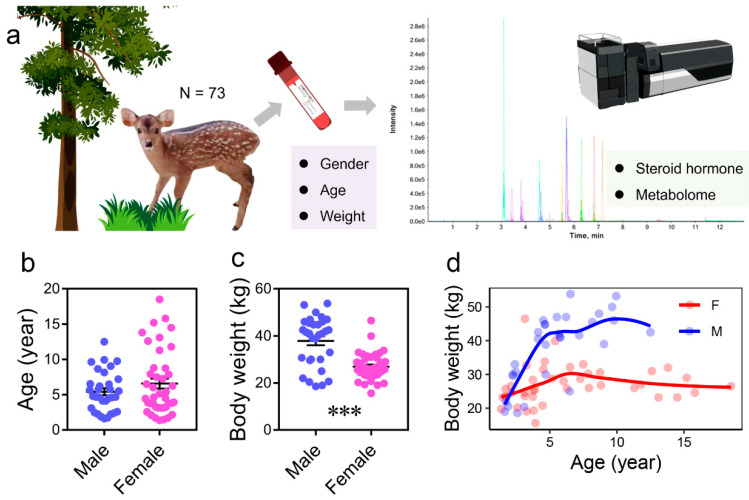
Experimental design and sample information. (**a**) Overview of the experimental design. Blood samples were collected from a total of 73 individuals for metabolomic analysis. (**b**,**c**) Body weight and age distribution of male and female individuals (***, *p* < 0.001). (**d**) Relationships between body weight and age across individuals.

The age structure of the sampled individuals showed no significant differences between sexes (Figure 1b and Table 1). Males exhibited significantly higher body weight than females (Figure 1b,c and Table 1). This difference is attributed to a plateau phase in weight growth observed in both sexes, with males reaching this plateau at a significantly higher body weight (*p* < 0.05) than females (Figure 1c).

### 3.2. Blood Hormone Profile of Hog Deer

Targeted metabolomics identified eleven steroid hormones in the blood of hog deer, including six sex hormones (estradiol, testosterone, estriol, dehydroepiandrosterone, stanolone, and progesterone) and four stress hormones (cortisol, cortisone, 11-deoxycortisol, and corticosterone) (Figure S1). Among these, estradiol was the most abundant, with concentrations reaching several micrograms per milliliter, a pattern consistent across both male and female individuals.

**Figure 2:**
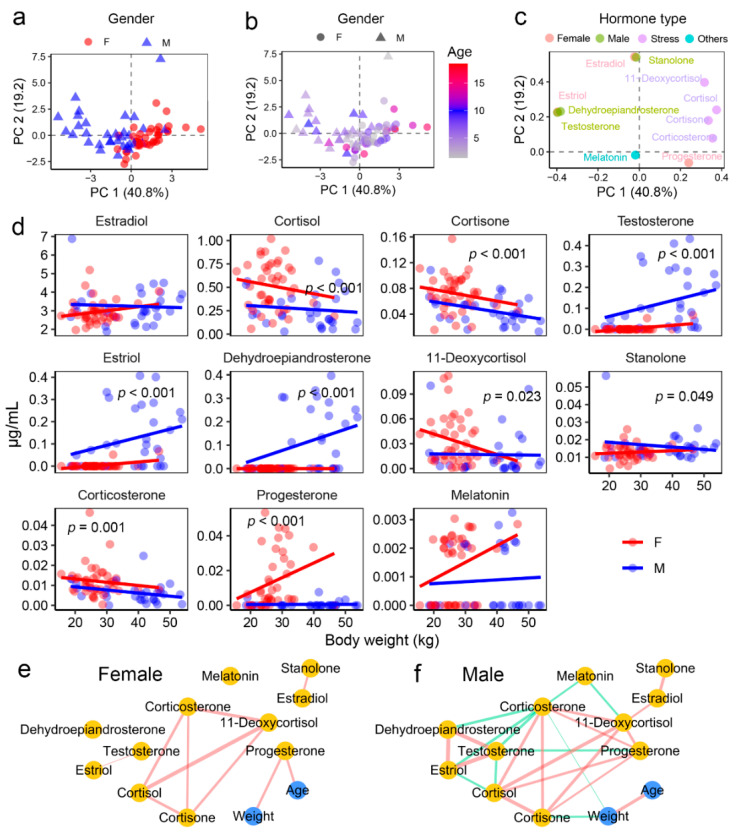
Hormone profiles in the blood of hog deer. (**a**) PCA scatter plot showing the differences in blood steroid hormone profiles between male and female individuals. (**b**) PCA scatter plot illustrating the dissimilarities in blood steroid hormone profiles between individuals with different ages. (**c**) Scatter loading plot highlighting the contributions of individual hormones to the PCA model, with dot colors representing hormone types. (**d**) Inter-sex differences in the abundance of each steroid hormone. (**e**,**f**) Network diagrams displaying quantitative correlations between physiological indices and metabolite abundance. Only pairwise correlations with an adjusted-*p* < 0.05 in Spearman correlations are shown. Edge colors represent correlation direction (red for positive and green for negative), and edge widths indicate the absolute R values.

The blood hormone profile effectively distinguished male and female samples (Figure 2a), while its variation with age or body weight was less pronounced (Figure 2b). Testosterone and progesterone were key contributors to the separation of sexes in the PCA model (Figure 2c). Further differential analysis (ANCOVA) identified hormones with significantly different levels between sexes. Females exhibited higher levels of progesterone and all four stress hormones, whereas males showed elevated levels of testosterone, dehydroepiandrosterone, and stanolone (Figure 2d and Table 2).

Correlation analyses indicated consistent relationships among the four stress hormones in both sexes (Figure 2e,f). In females, blood progesterone levels were strongly correlated with both age and body weight (Figure 2d and Figure S2), suggesting that progesterone could serve as a potential biomarker for age in this species. In males, however, no significant correlations were observed between physiological characteristics and hormone levels.

### 3.3. Blood Metabolic Profile of Hog Deer

**Figure 3:**
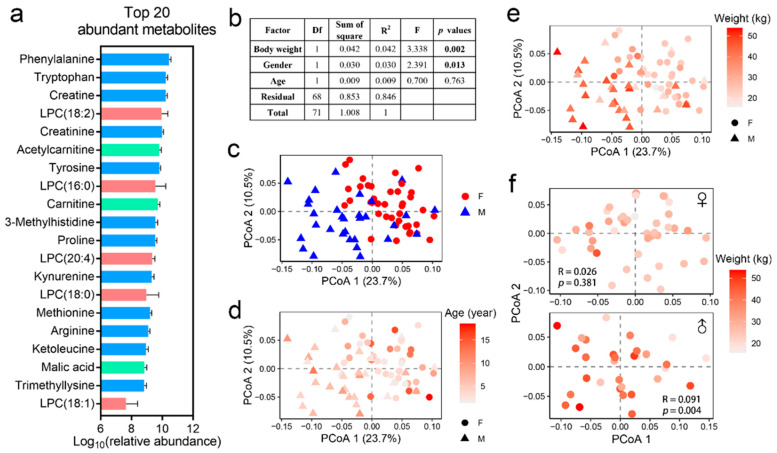
Metabolic profile of the blood. (**a**) Major features of the blood metabolome, represented by the 20 metabolites with the highest relative abundances (based on molecular ion peak area). Note that the relative peak area does not reflect the actual molar concentrations of the metabolites. (**b**) Effects of sex, body weight, and age on the blood metabolome of hog deer. (**c**–**e**) PCoA scatter plots illustrating the differences in the blood metabolome based on sex (**c**), age (**d**), and body weight (**e**). (**f**) PCoA scatter plots showing the variation in the blood metabolome with body weight, stratified by sex.

The most abundant metabolites identified in the blood of hog deer included amino acids (e.g., phenylalanine, tryptophan, proline, and tyrosine), lysophosphatidylcholines (LPCs), creatine, creatinine, and carnitine (Figure 3a). PERMANOVA analysis was performed to assess the influence of sex, age, and body weight on the overall metabolome. The blood metabolic profiles showed significant associations with sex and body weight, but not with age (Figure 3b–e). To account for potential sex-specific differences in the metabolome, the impact of body weight on the metabolic profile was further examined within each sex. Results showed that age-related variations in the metabolome were predominantly observed in males, while no such patterns were evident in females (Figure 3f).

**Figure 4:**
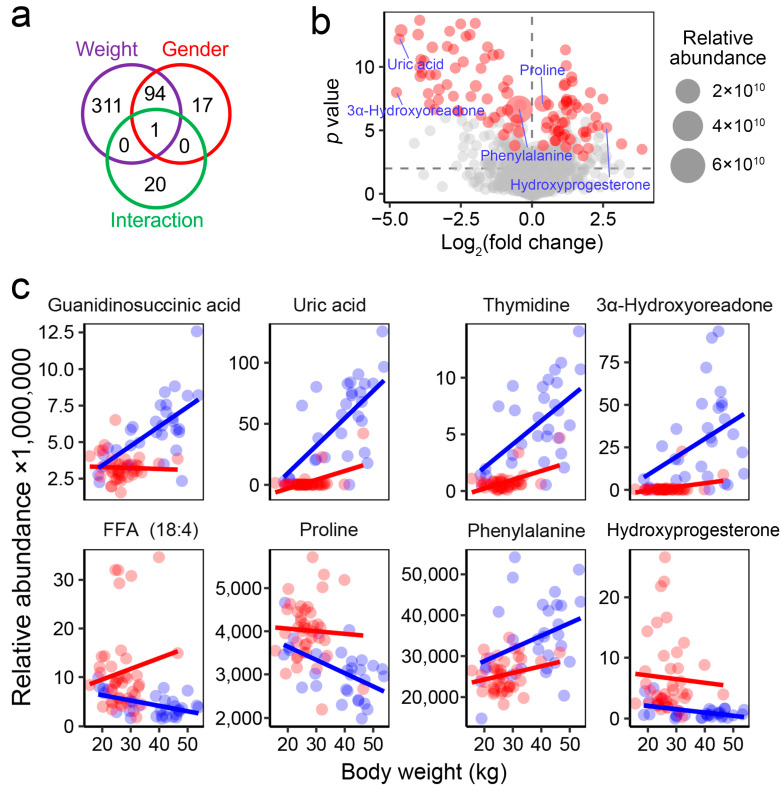
Metabolites showing sex-based differences in abundance. (**a**) Venn diagram displaying the number of differential metabolites (adjusted-*p* < 0.05) associated with body weight, gender, and their interaction. (**b**) Volcano plot illustrating fold changes and *p* values for metabolites between females and males. Each dot represents a metabolite; red dots indicate significant differences in abundance between groups (adjusted-*p* < 0.05). (**c**) Dot plots showing the relative abundances of eight representative metabolites with significant differences between sexes (red, female; blue, male).

Differential analyses (ANCOVA) were conducted to identify metabolites that differed significantly between sexes. A total of 112 metabolites were found to be sex-associated, with 94 of these also showing variations linked to body weight (Figure 4a). Among the identified differential metabolites, uric acid and 3α-hydroxyoreadone displayed the largest fold changes, while phenylalanine and proline showed the highest relative abundance (Figure 4b). Female individuals exhibited higher blood levels of free fatty acid (FFA) 18:4, proline, and hydroxyprogesterone, whereas males showed elevated levels of guanidinosuccinic acid, uric acid, thymidine, 3α-hydroxyoreadone, and phenylalanine (Figure 4c).

**Figure 5:**
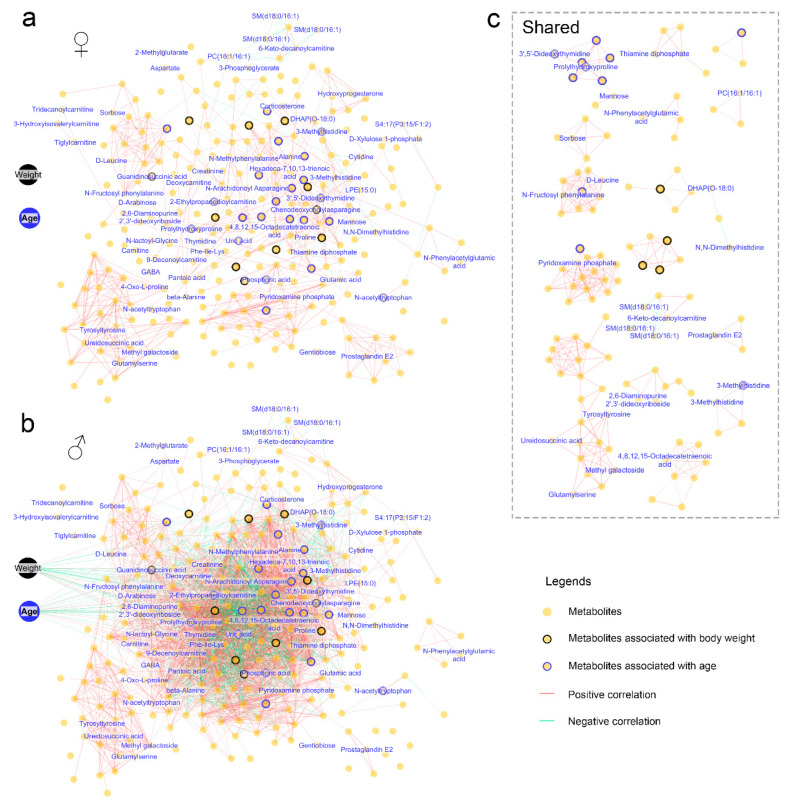
Correlation networks illustrating the associations between animal physiology and blood metabolic profiles. The networks display pairwise correlations with adjusted-*p* < 0.01 and absolute R values > 0.5. (**a**) Correlation networks in females. (**b**) Correlation networks in males. (**c**) Correlation networks shared by females and males. Each node represents a metabolite, with those bordered in black and blue indicating metabolites associated with body weight and age, respectively.

**Figure 6:**
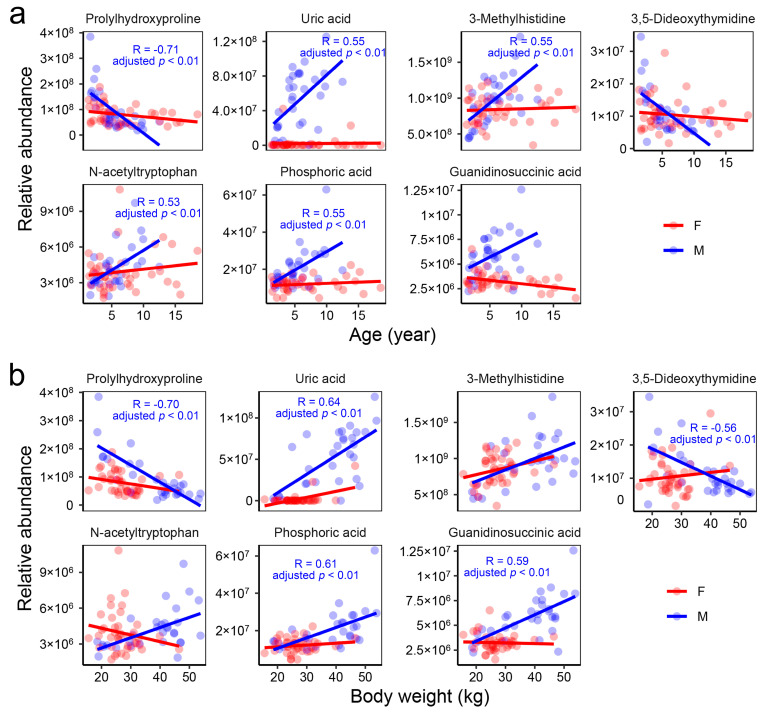
Potential blood biomarkers for age (**a**) and body weight (**b**) in male individuals. The text within the plots presents the statistical results for males.

The blood metabolome exhibited a more intricate correlation network in males compared to females (Figure 5a,b), with only a small portion of the correlations shared between the two genders (Figure 5c). Correlations between metabolites and the animals’ body weight and age were observed exclusively in males. Specifically, older males exhibited significantly (adjusted-*p* < 0.05) higher levels of uric acid, 3-methylhistidine, N-acetyltryptophan, and phosphoric acid, while showing lower levels of prolylhydroxyproline (Figure 6a). A higher body weight was associated with increased levels of uric acid, phosphoric acid, and guanidinosuccinic acid, as well as decreased levels of prolylhydroxyproline and 3,5-dideoxythymidine (Figure 6b). These metabolites may serve as potential blood biomarkers for age and body weight in males.

## 4. Discussion

**Figure 7:**
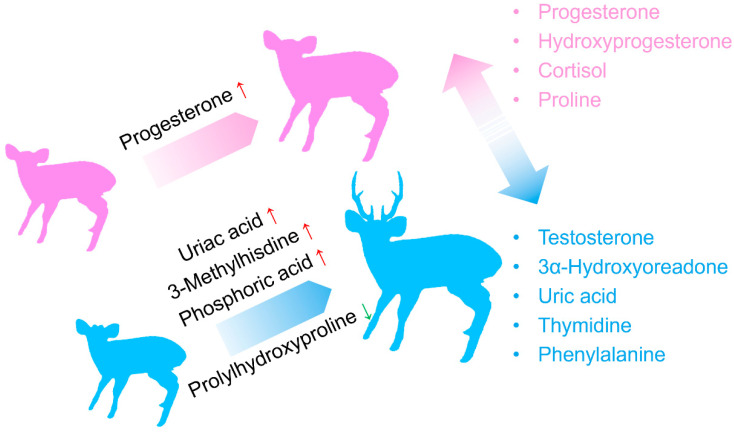
A graphical summary of the results, highlighting the potential blood biomarkers for sex and age/weight.

In this study, we present the largest blood metabolomics dataset to date for captive hog deer, comprising 73 individuals. Our findings reveal that progesterone and testosterone are the characteristic sex hormones for female and male hog deer, respectively. In addition to hormonal differences, we observed significant metabolic disparities between the sexes. Furthermore, we identified several metabolic biomarkers related to age and body weight in both female and male individuals (Figure 7). In the following discussion, we will explore the biological significance of these physiological markers and their potential conservation implications for muntjac breeding and rewilding initiatives.

### 4.1. Steroid Hormones in the Blood of Hog Deer

Estradiol is the dominant steroid hormone in the blood of hog deer, regardless of sex. Its concentrations can reach several μg/mL, which is much higher than the levels observed in humans and other mammals [21,22,23], indicating its significant role in hog deer physiology. Estradiol is widely recognized as the primary female sex hormone in mammals, involved in regulating female reproductive cycles, such as estrous and menstrual cycles [24]. In hog deer, male individuals exhibit estradiol levels comparable to those found in females. Although estradiol is traditionally considered to be present at much lower levels in males, recent evidence suggests that its concentrations can exceed those found in postmenopausal women, and some biological actions traditionally attributed to testosterone via the androgen receptor may, in fact, depend on its aromatization to estradiol [25]. Compared to other steroid hormones in hog deer, estradiol levels remain relatively stable among individuals and do not fluctuate significantly with endogenous factors such as age or sex. This stability makes estradiol a promising metabolic marker for identifying individuals with significant health issues or for studying the influence of exogenous factors on this species.

Progesterone and its analogue, hydroxyprogesterone, are key sex hormones in female hog deer. Progesterone, an endogenous steroid and progestogen, plays a critical role in the menstrual cycle, pregnancy, and embryogenesis in humans and other species [26]. Its levels serve as an important physiological marker for evaluating female reproductive health, particularly in predicting or enhancing reproductive potential and the likelihood of successful pregnancy [27,28,29]. Progesterone is not only a sexual biomarker, but also an indicator of age and weight in female hog deer (Figure 7). In females, progesterone levels may fluctuate after sexual maturation [30] and decrease with age or reproductive senescence [31]. Interestingly, older female hog deer in our study maintained higher progesterone levels, despite considerable individual variation. This could be promising for artificial population expansion efforts in hog deer. Testosterone is the primary sex hormone in male hog deer, reflecting sperm production, libido, reproductive behavior, and overall endocrine function [32,33]. Monitoring both progesterone and testosterone levels enables effective prediction and evaluation of reproductive potential, which could optimize breeding programs through selective parent breeding. Additionally, testosterone levels in males are associated with aggressiveness [34,35]. Given that fighting is a common cause of death in hog deer at Chengdu Zoo (personal communication), monitoring testosterone levels could help identify aggressive individuals and improve enclosure management.

A total of four steroid stress hormones were identified in hog deer, and all showed significantly higher concentrations in females than in males. Among them, cortisol is the primary stress hormone produced by the adrenal cortex. It plays a crucial role in the body’s response to stress, helping to regulate metabolism, inflammation, blood pressure, and immune function [36]. Gender-related differences in cortisol levels vary across mammalian species, influenced by reproductive status, stress exposure, and circadian rhythms [37]. Whether males or females have higher cortisol levels can vary depending on environmental factors. The consistently higher levels of stress hormones in female hog deer may suggest that they experience more significant physiological or psychological stress than the males. Therefore, the physical and mental well-being, as well as the housing conditions, of captive female hog deer may require more attention. Another interesting observation is the negative correlation between testosterone and stress hormone concentrations in male individuals. One potential explanation is that males with higher testosterone levels may experience lower stress due to their physiological advantages.

### 4.2. Metabolic Biomarker in the Blood of Hog Deer

The blood metabolomics data from 73 healthy hog deer individuals offer two significant implications for developing a health framework for this species. On one hand, it can serve as a valuable reference for identifying abnormal individuals, understanding underlying causes, and optimizing their daily care regimen. For example, an abnormal amino acid profile in the blood of certain individuals may indicate that the current diet is insufficient to meet their nutritional needs, necessitating targeted nutritional supplementation. On the other hand, variations in the metabolic profiles of healthy populations reveal biomarkers associated with physiological traits, such as gender, age, and body weight, which could be used to assess the population structure in wild individuals.

Phenylalanine and proline are among the most abundant amino acids in the blood of hog deer, displaying significant sexual dimorphism. These amino acids are essential for protein synthesis and metabolism, reflecting an individual’s nutritional status, metabolic health, and stress response. The sexual differences in their blood levels may indicate variations in nutritional requirements or metabolic patterns between genders.

Uric acid is recognized as a biomarker influenced by both gender and age/weight in hog deer (Figure 7). In humans, adult women typically have lower uric acid levels than age-matched men due to sex differences in renal clearance of uric acid [38], and these sexual differences in serum uric acid levels begin at puberty, partly due to the influence of muscle mass [39]. This may also explain the strong positive correlation between uric acid levels and body weight in male hog deer, whose body weight likely positively correlates with muscle mass. Uric acid has considerable clinical significance; elevated blood levels can lead to gout and are linked to other medical conditions, including diabetes and the formation of ammonium acid urate kidney stones [40]. Given that male hog deer have significantly higher blood uric acid levels than females, with the difference being much greater than in humans, it is important to investigate whether older males may be at increased risk for the negative effects associated with elevated uric acid levels. Since uric acid is a normal component of urine, exploring the relationship between urine uric acid levels and the physiological traits of hog deer could help develop non-invasive metabolic biomarkers.

3-methylhistidine serves as an age biomarker in male hog deer. This metabolite, a post-translationally modified amino acid, is considered a marker for skeletal muscle protein breakdown in humans [41]. The positive correlation between age and 3-methylhistidine levels may reflect an increased rate of muscle protein degradation in older males. Similar to uric acid, 3-methylhistidine is normally excreted in human urine, and it would be worthwhile to examine whether its urine levels correlate with the animal’s physiology.

In addition to the quantitative variations in metabolites, significant differences in the complexity of the correlation networks between females and males were also observed, with males exhibiting more pronounced pairwise correlations between metabolites in their blood. Since complexity generally enhances stability in a homeostatic system [42], our findings suggest that the blood metabolome of female hog deer may be more susceptible to external influences and, therefore, warrants closer attention in daily care.

It should be noted that there are some limitations in this study. First, the relationships between blood hormones, metabolites, and physiological traits of hog deer may be more complicated and always not straightforward. The results of our Spearman and Pearson correlation analyses may have led to the omission of important biomarkers or the identification of false positives. Therefore, additional mechanistic studies are needed to clarify the relationships between hormones, metabolites, and physiology in hog deer blood, and more data are required to validate the associations identified in this study. Second, the physiological traits and environmental factors considered in this study were limited. Certain physiological factors more directly linked to health, such as metabolic rate and heart rate, along with their associations with metabolism or hormones, would provide greater value for conservation biology. Third, the metabolomics data in this study were not absolutely quantified, which somewhat limits the ability to compare across studies.

## 5. Conclusions

This study presents the largest blood metabolomics dataset for hog deer, highlighting significant differences in hormone and metabolite profiles between sexes, along with potential biomarkers related to age and body weight. The identification of progesterone and testosterone as signature sex hormones, coupled with metabolic differences such as higher uric acid levels in males and proline levels in females, provides valuable tools for assessing health and informing breeding programs. These findings contribute to the development of a comprehensive health database that can support conservation and rewilding efforts for hog deer, offering critical insights into their physiological needs and potential conservation strategies. It is important to acknowledge some limitations in this study. Firstly, the untargeted metabolomics approach did not provide absolute quantification of metabolites, which limits the comparability of data across different sources. Additionally, the study lacks comprehensive physiological and environmental parameters for the animals, including phenotypic traits, environmental temperature, and the level of human interference. Future research should address these aspects.

## Figures and Tables

**Table 1:** Comparison of body weight and age between females and males. Data are presented as mean ± SD. Different superscripts indicate significant differences between groups.

Gender	Body Weight (kg)	Age (year)
Female (n = 41)	26.98 ± 5.66 ^a^	6.58 ± 4.57 ^a^
Male (n = 32)	37.87 ± 10.5 ^b^	5.42 ± 2.8 ^a^

**Table 2:** Hormone levels in the blood (mean ± SD, μg/mL). Asterisks denote significant differences between females and males: ***, *p* < 0.001; *, *p* < 0.05.

Hormones	Female	Male	Significance
Corticosterone	0.012 ± 0.008	0.006 ± 0.005	***
Cortisol	0.514 ± 0.249	0.263 ± 0.194	***
Cortisone	0.072 ± 0.027	0.045 ± 0.021	***
11-Deoxycortisol	0.033 ± 0.029	0.016 ± 0.025	*
Estradiol	2.938 ± 0.619	3.262 ± 0.965	n.s.
Estriol	0.003 ± 0.013	0.121 ± 0.13	***
Progesterone	0.013 ± 0.016	0.001 ± 0.002	***
Testosterone	0.003 ± 0.013	0.128 ± 0.134	***
Stanolone	0.013 ± 0.004	0.016 ± 0.008	*
Dehydroepiandrosterone	0 ± 0	0.113 ± 0.133	***
Melatonin	0.001 ± 0.001	0.001 ± 0.001	n.s.

## Data Availability

The authors will supply the relevant data in response to reasonable requests.

## Supplementary Materials

The following supporting information can be downloaded at: https://www.mdpi.com/article/10.3390/metabo15020126/s1, Supplementary Data S1 (pdf): Table S1, Sample information; Table S2, The hormone levels (μg/mL) in the blood; Figure S1, Hormone profile in the blood of hog deer (Mean ± SE); and Figure S2, Variations of blood hormone levels with the age of hog deer. Supplementary Data S2 (xlsx): Table S3, Metabolic profiles of untargeted metabolomics.

## Abbreviation

ANCOVA	Analysis of covariance
BH corrections	Benjamini-Hochberg corrections
EDTA	Ethylenediaminetetraacetic acid
FFA	free fatty acid
LPC	Lysophosphatidylcholine
MRM	Multiple reaction monitoring
PCA	Principal component analysis
PCoA	Principal coordinates analysis
PERMANOVA	Permutational multivariate analysis of variance
UHPLC	Ultra-high-performance liquid chromatography

## References

[1] Humphrey S.R. (2019). Endangered animals of Thailand.

[2] Duckworth W., Salter R.E., Khounboline K. (1999). Wildlife in Lao PDR: 1999 Status Report.

[3] Timmins R., Duckworth J.W., Samba Kumar N., Anwarul Islam M., Sagar Baral H., Long B., Maxwell A. (2015). Axis porcinus. The IUCN Red List of Threatened Species 2015. IUCN Red List. Threat. Species.

[4] Wang S. (1998). China Red Data Book of Endangered Animals-Mammalia.

[5] Wang Y.-X. (2003). A Complete Checklist of Mammal Species and Subsepceis in China: A Taxonomic and Geographic Reference.

[6] Lian H., Yu J.-Q., Ge Y.-F., Fang S.-G. (2009). Nine novel microsatellite markers for the hog deer (*Axis porcinus*). Conserv. Genet..

[7] Yu J.-Q., Wu K.-J., Li H.-W., Liu X.-Z., Mao J., Wang Q., Hoa K.-C., Chen H.-W. (2009). Preliminary study on the growth rule of young captive Hog deer. Sichuan J. Zool..

[8] Yu J.-Q., Liu X.-Z., Wang Q., Li H.-W., Wu K.-J. (2010). Feeding times and Amounts Given to Young Hog Deer (*Axis porcinus*) of 1~10 Weeks Reared in Captivity. Sichuan J. Zool..

[9] Deng J.-B., Yu J.-Q., Niu L.-L., Wang Q., Liu X.-Z. (2010). Determination of blood cell and biochemical indices on the captive Hog deer. Sichuan J. Zool..

[10] Angom S., Tuboi C., Ghazi M.G.U., Badola R., Hussain S.A. (2020). Demographic and genetic structure of a severely fragmented population of the endangered hog deer (*Axis porcinus*) in the Indo-Burma biodiversity hotspot. PLOS ONE.

[11] Geng G.-Y., You Y.-Y., Liu X.-Q. (2022). SSR Molecular Marker Development and Genetic Diversity Analysis of *Axis porcinus*. Chin. J. Widlife.

[12] Wang W., Yan H., Yu J., Yi J., Qu Y., Fu M., Chen A., Tang H., Niu L. (2017). Discovery of genome-wideSNPs by RAD-seqand the genetic diversity of captive hog deer (*Axis porcinus*). PLOS ONE.

[13] Yan H.J., Wang W., Yu J.Q., Yi J., Niu L.L., Chen H.W., Yu X. (2023). MAge-Related Changes in the Gut Microbiota Composition of Hog Deer (*Axis porcinus)*. Pakistan J. Zool..

[14] Maceda-Veiga A., Figuerola J., Martínez-Silvestre A., Viscor G., Ferrari N., Pacheco M. (2015). Inside the Redbox: Applications of haematology in wildlife monitoring and ecosystem health assessment. Sci. Total Environ..

[15] Melvin S.D., March D.T., Marshall K., Carroll A.R., van de Merwe J.P. (2021). Improving rehabilitation outcomes using metabolomics: Health, recovery and biomarkers of mortality in sick and injured green turtles (*Chelonia mydas*). Biol. Conserv..

[16] Liu Y., Liu F., Yu J.-Q., Niu L.L., Liu X.-Z., Li J. (2022). De novo Assembly Transcriptome of Blood in *Axis porcinus* Reveals Gender Differential Expression Genes. Sichuan J. Zool..

[17] Yan H.-J., Wnag W., Yi J., Niu L.-L., Qu Y., Chen A., Pu Y., Deng J.B., Zhong Y., Yu J.-Q. (2018). Seasonal Dynamics of Several Hormones in *Axis porcinus*. Sichuan J. Zool..

[18] Wang W., Yan H.-J., Chen S.-Y., Li Z.-Z., Yi J., Niu L.-L., Deng J.-P., Chen W.-G., Pu Y., Jia X. (2019). The sequence and de novo assembly of hog deer genome. Sci. Data.

[19] R Core Team (2021). R: A Language and Environment for Statistical Computing.

[20] Dixon P. (2003). VEGAN, a package of R functions for community ecology. J. Veg. Sci..

[21] Becker J.B., Berkley K.J., Geary N., Hampson E., Herman J.P., Young E. (2007). Sex Differences in the Brain: From Genes to Behavior.

[22] Depypere H.T., Bolca S., Bracke M., Delanghe J., Comhaire F., Blondeel P. (2015). The serum estradiol concentration is the main determinant of the estradiol concentration in normal breast tissue. Maturitas.

[23] Bubenik G.A., Miller K.V., Lister A.L., Osborn D.A., Bartos L., Van Der Kraak G.J. (2005). Testosterone and estradiol concentrations in serum, velvet skin, and growing antler bone of male white-tailed deer. J. Exp. Zool. Part A Comp. Exp. Biol..

[24] McCarthy M.M. (2008). Estradiol and the Developing Brain. Physiol. Rev..

[25] Russell N., Grossmann M. (2019). Mechanisms in Endocrinology: Estradiol as a male hormone. Eur. J. Endocrinol..

[26] Taraborrelli S. (2015). Physiology, production and action of progesterone. Acta Obstet. Et Gynecol. Scand..

[27] Wiltbank M.C., Souza A.H., Carvalho P.D., Cunha A.P., Giordano J.O., Fricke P.M., Baez G.M., Diskin M.G. (2014). Physiological and practical effects of progesterone on reproduction in dairy cattle. Animal.

[28] Filicori M. (2015). Clinical roles and applications of progesterone in reproductive medicine: An overview. Acta Obstet. Gynecol. Scand..

[29] Conley A.J., Gonzales K.L., Erb H.N., Christensen B.W. (2023). Progesterone Analysis in Canine Breeding Management. Vet. Clin. Small Anim. Pract..

[30] Lee S.J., Lenton E.A., Sexton L., Cooke I.D. (1988). The effect of age on the cyclical patterns of plasma LH, FSH, oestradiol and progesterone in women with regular menstrual cycles. Hum. Reprod..

[31] Hori K., Matsuyama S., Nakamura S., Iwata H., Kuwayama T., Miyamoto A., Shirasuna K. (2019). Age-related changes in the bovine corpus luteum function and progesterone secretion. Reprod. Domest. Anim..

[32] Muller M.N. (2017). Testosterone and reproductive effort in male primates. Horm. Behav..

[33] Deen A. (2008). Testosterone profiles and their correlation with sexual libido in male camels. Res. Vet. Sci..

[34] Turner A.K. (1994). Genetic and hormonal influences on male violence. Male Violence.

[35] Popma A., Vermeiren R., Geluk C.A.M.L., Rinne T., van den Brink W., Knol D.L., Jansen L.M.C., van Engeland H., Doreleijers T.A.H. (2007). Cortisol Moderates the Relationship between Testosterone and Aggression in Delinquent Male Adolescents. Biol. Psychiatry.

[36] Katsu Y., Baker M.E. (2021). Cortisol. Handbook of Hormones.

[37] Wang R., Kogler L., Derntl B. (2024). Sex differences in cortisol levels in depression: A systematic review and meta-analysis. Front. Neuroendocrinol..

[38] Mateos Antón F., García Puig J., Ramos T., González P., Ordás J. (1986). Sex differences in uric acid metabolism in adults: Evidence for a lack of influence of estradiol-17β (E2) on the renal handling of urate. Metabolism.

[39] Alvim R.O., Siqueira J.H., Zaniqueli D., Dutra D.M., Oliosa P.R., Mill J.G. (2020). Influence of muscle mass on the serum uric acid levels in children and adolescents. Nutr. Metab. Cardiovasc. Dis..

[40] Rock K.L., Kataoka H., Lai J.-J. (2013). Uric acid as a danger signal in gout and its comorbidities. Nat. Rev. Rheumatol..

[41] Elia M., Carter A., Bacon S., Winearls C.G., Smith R. (1981). Clinical usefulness of urinary 3-methylhistidine excretion in indicating muscle protein breakdown. Br. Med. J..

[42] Waldrop M.M. (1993). Complexity: The Emerging Science at the Edge of Order and Chaos.

[43] Percie du Sert N., Ahluwalia A., Alam S., Avey M.T., Baker M., Browne W.J., Clark A., Cuthill I.C., Dirnagl U., Emerson M. (2020). Reporting animal research: Explanation and elaboration for the ARRIVE guidelines 2.0. PLoS Biol..

