# Supplementary material for: Physiology-Related Variations in the Blood Hormone and Metabolome of Endangered Hog Deer (Axis porcinus)
Source: Metabolites. 2025 Feb 13;15(2):126. doi: 10.3390/metabo15020126 (PMC11857704; doi:10.3390/metabo15020126)
Supplement: Supplementary file 1 [file metabolites-15-00126-s001.zip › Supplementary data 1.pdf]

Tables

Table S1 Sample information.

Sample ID	Gender	Body weight (kg)	Age (years)
AP-1	M	44.8	4.64
AP-2	M	40.5	4.13
AP-3	M	41	5.77
AP-4	M	\	4.79
AP-5	M	47	5.53
AP-6	M	47	6.21
AP-7	F	46.5	3.15
AP-8	M	25	6.48
AP-9	M	53.2	9.95
AP-10	M	22	1.73
AP-11	F	33	7.49
AP-12	M	46	4.52
AP-13	F	23.2	13.1
AP-14	M	19	1.64
AP-15	F	24.6	3.15
AP-16	F	19.8	1.38
AP-17	M	42.5	12.5
AP-18	F	26.6	2.12
AP-19	F	23.2	14.5
AP-20	M	45.5	8.16
AP-21	M	53.8	6.54
AP-22	F	25.7	1.7
AP-23	F	26	7.92
AP-24	F	25.6	3.89
AP-25	M	30.7	2.46
AP-26	M	20.4	3.09
AP-27	F	28.8	5.17
AP-28	F	28.7	7.27
AP-29	F	39.9	5.4
AP-30	F	31.5	4.52
AP-31	F	30.6	8.75
AP-32	F	32.2	6.51
AP-33	F	26	11.3
AP-34	F	32.7	3.26
AP-35	M	20.6	1.89
AP-36	M	18.6	2.59
AP-37	F	19.3	3.8
AP-38	F	20.8	4.46
AP-39	F	19.05	3.27
AP-40	M	46	9.77

AP-41	F	26.5	18.5
AP-42	F	15.6	3.93
AP-43	F	27	11.8
AP-44	M	30	2.24
AP-45	F	31	12.6
AP-46	M	42.5	4.13
AP-47	M	38.8	4.69
AP-48	F	23.6	3.13
AP-49	M	33	2.4
AP-50	M	41	5.77
AP-51	F	24.6	15.8
AP-52	F	24.6	2.17
AP-53	F	28.4	3.74
AP-54	F	30.2	7.86
AP-55	F	23.2	1.48
AP-56	M	44.8	4.64
AP-57	F	23.5	1.71
AP-58	M	29.6	2.26
AP-59	M	30	4.69
AP-60	F	33.8	4.63
AP-61	F	25.2	3.77
AP-62	M	41.6	7.19
AP-63	F	26.8	8.81
AP-64	F	32	13.8
AP-65	F	23.4	3.73
AP-66	F	29	15.2
AP-67	F	20.2	2.58
AP-68	F	25.8	6.27
AP-69	F	28	6.03
AP-70	M	39	9.04
AP-71	M	50	9.65
AP-72	M	42	5.67
AP-73	M	48	8.67

Table S2 The hormone levels (μg/mL) in the blood.

**Sample ID**	**Corticosterone**	**Cortisol**	**Cortisone**	**11-Deoxycortisol**	**Estradiol**	**Estriol**	**Progesterone**	**Testosterone**	**Stanolone**	**Dehydroepiandrosterone**	**Melatonin**
**AP-1**	0.003038987	0.1609606	0.03966139	0.0038245	4.45666996	0.28517223	0	0.27738892	0.01642165	0.282616557	0.00324561
**AP-2**	0.003009628	0.42119836	0.06606282	0.0274297	4.25387082	0.40658123	0	0.40987619	0.02201686	0.333726003	0.00280728
**AP-3**	0.002616262	0.22583266	0.03792036	0.01264788	3.92936146	0.27617802	0	0.29455559	0.02233105	0.308049889	0
**AP-4**	0.002819496	0.14983668	0.03034696	0.00390107	3.77935753	0.07017695	0	0.07280781	0.01591767	0.102053817	0
**AP-5**	0.006881379	0.37693572	0.04329712	0.0159138	3.5330807	0.03516452	0	0.04135794	0.01697854	0.063560342	0
**AP-6**	0.008973951	0.66508827	0.05621383	0.04068759	3.98923713	0	0	0.00947794	0.01205985	0	0
**AP-7**	0.007739053	0.18890379	0.04439941	0.00359517	4.0525912	0.06686158	0	0.06746354	0.01451098	0	0.0028415
**AP-8**	0.008590095	0.11651161	0.03252683	0.0038056	3.92714099	0.07221531	0	0.06379127	0.01806627	0	0.00215579
**AP-9**	0.00540357	0.05174047	0.01299899	0.0041113	3.63479861	0.24288164	0	0.26453539	0.01581198	0.251748675	0
**AP-10**	0.004660157	0.21689715	0.03480129	0.00294615	3.49782862	0	0	0	0.0100511	0	0
**AP-11**	0.013460132	0.68023328	0.07037112	0.04601725	3.61884546	0	0.0033707	0	0.01354839	0	0
**AP-12**	0.000504252	0.12586442	0.04048205	0	3.26719938	0.07713396	0	0.08116101	0.01505112	0	0.00220166
**AP-13**	0.022229708	0.84922705	0.04974569	0.10875817	4.35772872	0	0.00727142	0	0.01614137	0	0.00228351
**AP-14**	0.013059042	0.56475476	0.10837906	0.10001976	6.87474825	0	0.00445089	0	0.0563722	0	0
**AP-15**	0.008846553	0.88174149	0.05762384	0.09524957	5.19204918	0	0.00374139	0	0.02148362	0	0.00181302
**AP-16**	0.006932022	0.50995412	0.09238131	0.04212273	3.0440342	0	0	0	0.00992053	0	0.00230531
**AP-17**	0.000759194	0.06831677	0.02966982	0.00027273	2.94409625	0.09653069	0	0.09998503	0.01100673	0.133463878	0.00190471
**AP-18**	0.008389624	0.75622227	0.08129733	0.04084302	2.97799419	0	0.00268957	0	0.0154652	0	0.00303372
**AP-19**	0.012494448	0.88327625	0.07929489	0.06035264	2.78653203	0	0.00839719	0	0.0114777	0	0.00199643
**AP-20**	0.00191666	0.13995832	0.03755169	0.00569633	3.13341752	0.40055159	0	0.43256765	0.01571137	0.396196818	0.00224642
**AP-21**	0.000591305	0.12392538	0.02969544	0.00852232	3.8939631	0.2104332	0	0.21025269	0.01792555	0.219129679	0
**AP-22**	0.004009679	0.32828168	0.07605318	0.00073081	3.92852729	0	0.01481706	0	0.02591379	0	0.00188948
**AP-23**	0.003545091	0.36439273	0.03807916	0.00997446	3.01220586	0	0	0	0.00507984	0	0.00182287

**AP-24**	0.004521338	0.37143178	0.07644608	0.02158659	3.29674921	0	0.00407525	0	0.01005468	0	0.00242763
**AP-25**	0	0.02689253	0.01605211	0.00258297	3.11037315	0.30821152	0	0.31950066	0.01073452	0.30375531	0.00205101
**AP-26**	0.010989168	0.27646907	0.05961211	0.01220947	3.28719876	0.04918158	0	0.04712756	0.01675124	0	0
**AP-27**	0.01647665	0.78495467	0.094153	0.04851863	3.40166245	0	0.03882632	0	0.01020728	0	0.00175736
**AP-28**	0.000392388	0.61822057	0.09292183	0.01753626	2.92566529	0	0.00287748	0	0.01896461	0	0.00203631
**AP-29**	0.007799476	0.3354372	0.05347555	0.00970675	3.39346983	0	0.03340282	0	0.01729318	0	0.00198549
**AP-30**	0.011662367	0.2673461	0.06398182	0.01409753	3.15472152	0	0.01806551	0	0.01320171	0	0.00273638
**AP-31**	0.010672428	0.25483093	0.05614403	0.01038318	2.87591448	0	0.0269371	0	0.01471226	0	0
**AP-32**	0.012245678	0.71052043	0.05216721	0.04256887	2.71261524	0	0.04025034	0	0.01008741	0	0.0027399
**AP-33**	0.007586748	0.36901021	0.06164716	0.01623707	2.56991958	0	0.01336822	0	0.0134266	0	0.00220922
**AP-34**	0.011138533	0.43484845	0.07460515	0.02265397	2.64194104	0	0.02355047	0	0.01133613	0	0
**AP-35**	0.00847274	0.20355642	0.05545291	0.01043082	2.95618853	0	0	0.01240446	0.01277989	0	0
**AP-36**	0.013330046	0.5294291	0.06684536	0.01882951	2.66753004	0	0	0.0135952	0.00984761	0	0
**AP-37**	0.007912626	0.31133319	0.06585882	0.01056562	3.4364931	0	0	0	0.01721864	0	0
**AP-38**	0.013929646	0.24984684	0.06525922	0.01459495	2.76220447	0	0	0	0.01480501	0	0
**AP-39**	0.014765613	0.73792895	0.07690571	0.05605659	2.88320286	0	0.0064887	0	0.01534501	0	0
**AP-40**	0.006368696	0.05363303	0.01965345	0	2.67970486	0	0	0.01055591	0.00948636	0	0
**AP-41**	0.018568511	1.0185078	0.08709485	0.06577842	2.48847552	0	0.00346456	0	0.01136551	0	0
**AP-42**	0.01564897	0.67141054	0.11118491	0.04168184	2.72573968	0	0	0	0.01080159	0	0
**AP-43**	0.007300759	0.11522749	0.02206034	0.0014564	2.90537399	0	0.02210321	0	0.01208781	0	0
**AP-44**	0.007932702	0.1907214	0.05475518	0.00212471	2.9027448	0.09515318	0	0.10015456	0.01487008	0	0.00215139
**AP-45**	0.030489233	0.89314017	0.10935507	0.06778622	2.629617	0	0.04400803	0	0.01463079	0	0.00276606
**AP-46**	0.0066985	0.05511985	0.02025007	0.00115089	2.13106642	0.12161528	0	0.12104462	0.01064439	0.119938301	0.00202259
**AP-47**	0.007435249	0.22613615	0.04182812	0.01059244	2.33719827	0	0	0.01372253	0.01628678	0	0
**AP-48**	0.012656643	0.4198025	0.05854384	0.01543472	2.64036899	0	0	0	0.01611216	0	0.00226814

**AP-49**	0.012618667	0.38003476	0.05376938	0.01967523	2.65792585	0.10298916	0	0.09909902	0.01200351	0.112486615	0
**AP-50**	0.005680267	0.31135049	0.07940523	0.01585671	2.71091144	0	0	0.0100689	0.01332127	0	0.00218201
**AP-51**	0.046322259	0.71189376	0.09763925	0.11197102	2.41366576	0	0.05331203	0	0.00781398	0	0
**AP-52**	0.010758116	0.32627883	0.07909024	0.02221758	2.23683571	0	0	0	0.01526882	0	0
**AP-53**	0.016451156	0.75676227	0.06856234	0.05925795	2.47173279	0	0.01011267	0	0.01256961	0	0
**AP-54**	0.014700267	0.58643215	0.09982238	0.03029731	2.37653397	0	0.03084906	0	0.01374951	0	0.00214917
**AP-55**	0.010253323	0.41576394	0.1063482	0.01604823	2.56588554	0	0	0	0.01062161	0	0.00200954
**AP-56**	0.002894184	0.15646649	0.02915437	0.00252569	2.20083359	0.19664121	0	0.20749949	0.01016384	0.225235101	0
**AP-57**	0.009039287	0.59309069	0.06546033	0.04338106	2.64110811	0	0.03690386	0	0.01135071	0	0
**AP-58**	0.006034921	0.34245304	0.05429378	0	2.15220019	0	0	0.00653512	0.01501744	0	0.0021492
**AP-59**	0.004137778	0.20549768	0.03195593	0.00227765	1.87792485	0.33287532	0	0.34884324	0.01147813	0.305585598	0
**AP-60**	0.007229737	0.4900366	0.07425419	0.0147668	2.63584073	0	0.00337657	0	0.0097863	0	0
**AP-61**	0.018640304	0.67486315	0.09161946	0.0529982	3.0346288	0	0.00248423	0	0.01693718	0	0
**AP-62**	0.02472049	0.78363118	0.07657972	0.05940594	2.93702563	0	0.00759913	0	0.01249344	0	0
**AP-63**	0.014689715	0.59621329	0.09332572	0.02616927	2.27508022	0	0.00871507	0	0.01109487	0	0
**AP-64**	0.008057004	0.15761188	0.0201196	0.00395713	2.72854221	0.05164206	0.00385218	0.05308526	0.01248983	0	0.00199419
**AP-65**	0.006981095	0.18395905	0.03875966	0.00991258	2.07208751	0	0	0	0.00595188	0	0.00231293
**AP-66**	0.005181513	0.23360198	0.04384146	0.00159006	2.6479748	0	0.04469362	0	0.0087182	0	0.00233701
**AP-67**	0.013157407	0.05701809	0.01571708	0.00888444	1.96354044	0	0	0	0.00358868	0	0
**AP-68**	0.013523412	0.73339169	0.15705292	0.06234286	2.93503268	0	0.00450971	0	0.01254349	0	0.00245389
**AP-69**	0.00794133	0.56526162	0.09135721	0.00915282	3.0532725	0	0.03255773	0	0.00794463	0	0.00232059
**AP-70**	0.004524372	0.1637128	0.05636257	0.00709061	2.21562648	0.25971714	0	0.28123041	0.01303847	0.253318718	0
**AP-71**	0.010789537	0.46002722	0.06494218	0.09584895	2.96123198	0.16300513	0.00324161	0.17528152	0.01453076	0.191421664	0
**AP-72**	0.002783161	0.09178109	0.01887893	0	2.98863193	0.06330863	0	0.04904078	0.02039768	0	0.00214239
**AP-73**	0.005511029	0.54845853	0.03232412	0.03600944	4.50162177	0	0.00237251	0.00684188	0.02650353	0	0

Figures

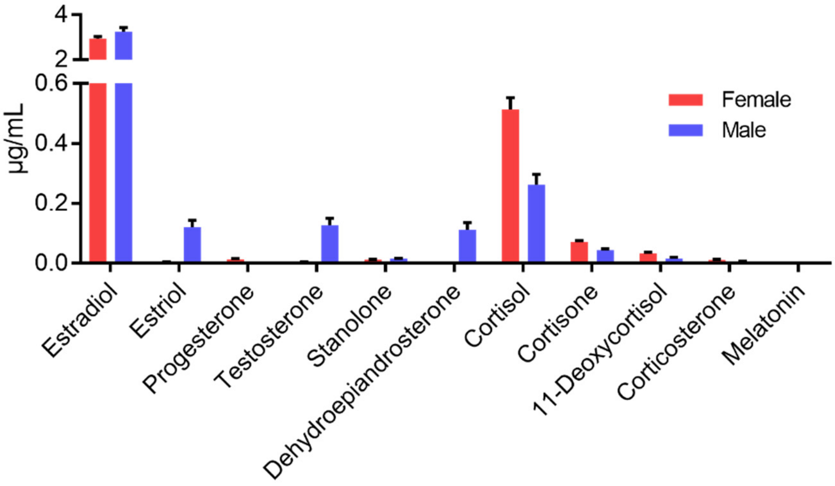

Figure S1 Hormone profile in the blood of hog deer (Mean ± SE).

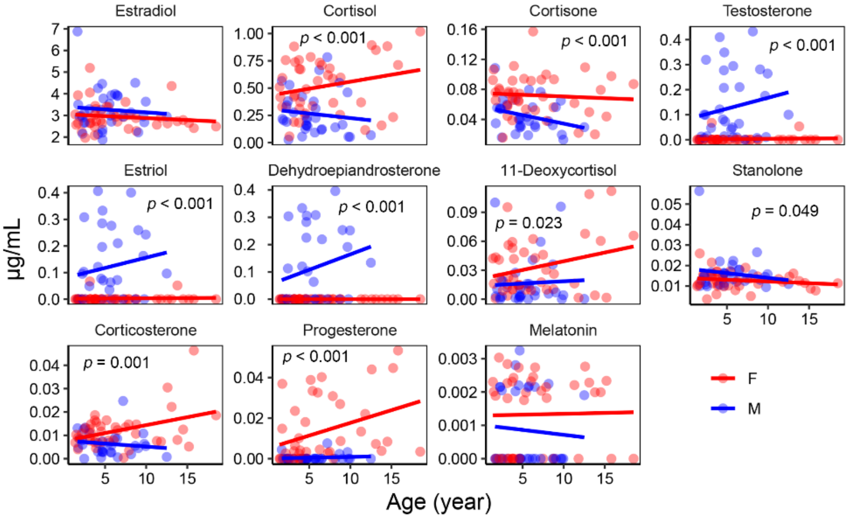

Figure S2 Variations of blood hormone levels with the age of hog deer.
